# Induction of Apoptosis by *Acanthaster planci *sp., and *Diadema setosum* sp., Fractions in Human Cervical Cancer Cell Line, HeLa

**DOI:** 10.31557/APJCP.2021.22.5.1365

**Published:** 2021-05

**Authors:** Gul-e-Saba Chaudhry, Murni Islamiah, Muhammad Naveed Zafar, Kamariah Bakar, Nur Asniza Aziz, Jasnizat Saidin, Yeong Yik Sung, Tengku Sifzizul Tengku Muhammad

**Affiliations:** 1 *Institute of Marine Biotechnology, Universiti Malaysia Terengganu, Kuala Terengganu, Malaysia. *; 2 *Department of Chemistry, Quaid-i-Azam University, Islamabad, Pakistan. *

**Keywords:** Apoptosis, phosphatidylserine externalization, DNA fragmentation, Acanthaster planci, Diadema setosum

## Abstract

Cancer is an uncontrolled multiplication of cells. The desire efficacy and severe toxicity of current anticancer drugs urge exploring and investigating a better alternative to existing chemotherapeutics. Natural products of marine origin are excellent sources of potential new drugs of enhanced biological activities.

**Objectives:**

Thus, the cytotoxic effects along with investigating the mode of cell death exerted by fractions, AP-9, AP-THR, DS-8 and DS-9 fraction of *Acanthaster planci*, Diadema setosum sp., on the human cervical cancer cell line, HeLa.

**Methods:**

The cytotoxicity of fractions has determined by using an MTS assay. The early and late apoptosis was studied by using the High content Screening (HCS) instrument.

**Results:**

The four fractions produced effective cytotoxicity effects with IC_50_ values at 72hr of less than 20 μg/ml in the order of AP-9 > DS-9 > APTHR-9 > DS-8. The fraction s exhibited cytotoxicity via mediating apoptotic mode of cell death. The early apoptosis by exposure of phosphatidylserine to the outer leaflet of the plasma membrane and late apoptosis due to the presence of green stain (DNA fragmentation) in treated cells.

**Conclusion:**

The potent bioactive compounds might be responsible for inducing apoptosis in cancer cells and, thus, the potential to be a successful candidate for exploring upcoming chemotherapeutic drugs.

## Introduction

Cancer is amongst the baneful diseases globally; of the estimated more than 570 000 deaths from cervical cancer every year, more than 84% of these occur in less developed regions (WHO 2019). Cancer is a complex disease that initiates when cells in the body start to grow uncontrolled and abnormal (Chaudhry et al., 2021; Chaudhry et al., 2021). The significant issues of current chemotherapy are low efficacy and severe toxicity, which encourages new findings to identify new potential and safe anticancer agents endowed with improved pharmaco-toxicological properties. In able to do so, emerging natural compounds could be an effective candidate, which plays a vital role in causing less toxicity and improved efficacy (Amin et al., 2009).

Largely, agents that induce apoptosis considered as potential anticancer therapeutics (Wu et al., 2015; Lee et al., 2015). Apoptosis is a natural mechanism for death called programmed cell death play a critical role in the growth and developmental process. The cells undergoing apoptosis reveal characteristic features, such as condensation of chromatin material, cytoplasm shrinkage, phosphoserine exposure and DNA fragmentation (Alisonand et al., 1992; Cotter et al., 1990).

Marine organisms are an abundant source of novel distinct natural products having potential pharmacological activities. Some products obtained from marine sources are currently in the preclinical and clinical trials phase to screen a potent chemotherapeutic agent (Schumacher et al., 2011; Wang et al., 2006). However, the major part of marine potential remains unknown and need to explore in the development of anticancer drugs. Various pharmacological studies showed that sea urchin extracts have biological effects, such as anticancer (Zhang 2002). The compounds isolated from sea urchin able to offer the antiproliferation activity, such as sulfoquinovosylmonoacyl- glycerol extracted from sea urchin, could inhibit cancer cells (Sahara et al., 1997). Polysaccharides extracted by the egg of Strongylocentrotus nudus exhibited antitumor activity mediated by the activation of TLR2/4 and induced cytotoxicity in NK. cells in-vivo (Ke et al., 2014). 

Starfish belongs to the class Asteroidea, phylum Echinodermata; around 1500 species are generally distributed in oceans worldwide. Various studies signified that starfish possessed active pharmacological and biological features. Amongst different spices, Oreaster reticulates, Luidia senegalensis and Echinaster used traditional medicine for asthma, bronchitis, diabetes and heart diseases in Brazil (Alves and Rosa 2007; Alves and Rosa 2011). However, the secondary metabolites isolated from starfish have received much attention, such as ceramide and cerebrosides (Inagaki et al., 2006; Ishii 2006). The metabolites isolated from starfish might categorised into three groups 1) asterosaponins, 2) cyclic steroidal glycosides, and 3) polyhydroxylated glycosides steroids. Glycosides belong to the asterosaponins, exhibit various biological activities, including hemolytic, cytotoxic, antifungal, antibacterial and antiviral activities (Tang et al., 2006). Cerebrocides metabolites isolated from starfish possess cytotoxic activity; induce apoptosis and potential anticancer properties (Zuo et al., 2013).

The exploration of active agents against cancer in the various marine organism has revealed numerous active compounds. In this paper, our interest is echinoderms, sea urchins, and starfish. Echinoderm produces several potential natural products. However, extensive research is mandatory to define their cytotoxicity and mode of cell death. Therefore, in this study, starfish sp., *Acanthaster planci* and sea urchin sp., *Diadema setosum* fractions selected to evaluate their cytotoxicity activity and mode of cell death on human cervical cancer (HeLa) cell line. 

## Materials and Methods

*Materials*

The human cervical cancer cell line, HeLa cell line, was purchased from ATCC, American Type Cell Culture, USA. For imaging analysis, ApoAlertTM Annexin V, purchased from Clontech, U.S.A., DeadEnd™ Fluorometric TUNEL and growth inhibition assay using M.T.S., CellTiter 96 Aqueous Non-Radioactive Cell Proliferation Assay, obtained from Promega USA. The reagents used purchased from Sigma Aldrich, U.S.A. All the chemicals used were of analytical grade. 

*Acanthaster planci and Diadema setosum Sp., sample collection and preparation*

The fractions of *Acanthaster planci* and *Diadema setosum* prepared using the Medium pressure liquid chromatography (MPLC) method previously reported (Gul-e-Saba et al., 2018). The specimens encoded (ECH1017002; ECH1017003) deposited in the Institute of Marine Biotechnology, UMT, Malaysia. Samples collected from Bidong Island, Terengganu with coordinate (5˚37’12 N 103˚3’48 E) as shown in Table 1. 

*Cytotoxicity activity of fractions*

Growth inhibitory or cytotoxic effects of fractions of *Acanthaster planci* and *Diadema setosum* on HeLa cell line were determined using CellTiter 96TM AQueous Non-Radioactive Cell Proliferation Assay (Chaudhry et al., 2020; Chaudhry et al., 2020; Gul-e-Saba et al., 2019; Muhammad et al., 2017). Briefly, the HeLa cells were seeded at a density of 2 × 10^4^ cells/well cells in 96 well plates and incubated at 37^o^C in a 5% CO_2_ incubator. After treatment, 20μl of MTS reagent was added and incubated for 3 hr. The absorbance was then read at a wavelength of 490 nm using an ELISA reader (Multiskan, Thermo Fischer U.S.A.). Cytotoxicity experiments carried out in triplicates, and results expressed as percentage growth inhibition of control. IC_50_ values for growth inhibition was derived from a nonlinear regression model (curve fit) based on a sigmoidal dose-response curve (variable) and computed using GraphPadPrism (GraphPad). Data has given as mean ± SEM ANOVA *P<0.05, **P<0.01 (Dunnett post-test).

*Early apoptosis (Phosphatidylserine exposure) in treated cells*

The Annexin V-FITC Apoptosis Detection Kit (APO Alert Annexin V, USA) used to determine the exposure of phosphatidylserine (PS) exerted by the fractions on the HeLa cells (Maher et al., 2019; Chaudhry et al., 2019). The cells were seeded at a cell density of 10,000 cells/96-well plate and incubated at 37^o^C for 24hr. The old medium was then replaced with a fresh medium containing the concentration of IC_50_ at 72hr of each fraction individually and incubated at 3hr, 6hr and 24hr, respectively. Next, cells were washed with 1× binding buffer (twice) and incubated in 200μL of binding buffer containing annexin V-FITC and propidium iodide (PI) at 37°C for 5-15 mins. Vincristine sulfate (drug) used as the positive control. The plates observed under ImageXpress Micro XLS. Widefield High-Content Analysis System (HCS) (Sunnyvale, USA), for images.

*Late apoptosis (DNA Fragmentation) in treated cells*

The DeadEndTM Fluorometric Apoptosis Detection System (Promega, USA) has used to analyze the DNA fragmentation induced cell death (apoptosis) in treated HeLa cells as our previously reported methods (Chaudhry et al., 2020; Zafar et al., 2019). The HeLa cells were cultured in Chamber Slides (Nunc, Denmark) with a density of 10,000 cells in each chamber and incubated at 37oC in a humidified condition of 5% CO_2_. The green fluorescence of fragmented nicked genomic DNA represents apoptotic cells detected by Image Xpress Micro XLS. Widefield High-Content Analysis System (HCS) (Sunnyvale, USA). 

## Results

The growth inhibition or cytotoxicity effects of four fractions, AP-9, APTHR-9, DS-8 and DS-9 fractions prepared from *Acanthaster planci*, *Diadema setosum* on human cervical cancer HeLa cell line investigated. 

The AP-9 fraction of *Acanthaster planci* sp., inhibited the growth of cells at concentrations 0.937 μg/ml at 24hr, 48hr and 72hr (Figure 1A). At 24hr and 48hr, lesser concentrations of fraction, 1.87, 3.75 and 7.5 μg/ml unable to show any inhibition in the growth of HeLa cells. Interestingly, the fraction produced a potent cytotoxicity effect on HeLa cell line at concentrations 0.937 μg/ml, where 60% of cell growth inhibition observed at 72hr. The IC_50_ values decreased as the incubation periods increased from >60 μg/ml (24hr) to 39.81 μg/ml (48hr) and 3.16 μg/ml (72hr).

A similar pattern of growth inhibition observed in APTHR-9 fraction of *Acanthaster planci* (thorn) sp. At 24hr, lesser concentrations unable to reduce the growth, only 60μg/ml inhibited more than 25% of growth inhibition at 24hr incubation. Interestingly, the fraction significantly inhibited the growth of HeLa cells at concentrations of 0.937 μg/ml and above after 48hr and 72hr. Similarly, at the concentration of 3.75μg/ml, 20% and 23% growth inhibition was noticed at the same incubation period (Figure 1D). However, the fraction exhibited cytotoxicity activity against the HeLa cell line with IC_50_ values of 28.79μg/ml (48hr) and 15.84μg/ml at 72hr treatment.

Similarly, the DS-8 fraction of *Diadema setosum* sp., also produced a growth inhibitory activity on the HeLa cell line. At 24hr incubation, the percentage of cell growth significantly inhibited at concentrations of 0.937 μg/ml after 24hr, 48hr and 72hr. Moreover, at a concentration of 3.75, there was significant inhibition in cell growth with 20% and 65% after 48hr and 72hr, respectively. Interestingly, 1.875μg/ml concentration reduced 63% of cell growth after 72hr incubation (Figure 1B). Furthermore, the fraction exhibited significant cytotoxicity activity against the HeLa cell line with IC_50_ values of 20.33 μg/ml (48hr), and 18.93 μg/ml (72hr). 

The DS-9 fraction of *Diadema setosum* sp., inhibit the growth of HeLa cells in a dose-dependent manner for 24hr, 48hr and 72hr. The significant inhibition in the growth of HeLa cells observed at concentrations 7.5μg/ml and above, after 24hr, 48hr, and 72hr incubation (Figure 1C). Moreover, at 7.5μg/ml 50%, 76% and 60% of growth inhibition noticed after 24hr, 48hr and 72hr incubation. Interestingly, at lesser concentration of 1.875, 21% (48hr) and 65 % (72hr) were recorded. The IC_50_ of fraction were 6.13 μg/ml and 3.36 μg/ml at 24hr and 48hr. However, the increase in IC_50_ values after 72hr, which was 6.89 μg/ml. Hence, the lower IC_50_ recorded after 48hr DS-9 fraction treatment incubation. Vincristine sulphate used as a positive control in our study showed the IC_50_ value of 0.005 μg/ml at 72 hr.

Furthermore, fractions of *Acanthaster planci* and *Diadema setosum* has shown cytotoxicity effects on the human cervical cancer cell line, HeLa (Table 1). The relative potential of cytotoxicity of the fractions at 72hr as follows: *Acanthaster planci* AP-9 > *Diadema setosum* DS-9 > *Acanthaster planci* (Thorn) APTHR-9 > *Diadema setosum* DS-8.

To analyze the mode of cell death induced by fractions of *Acanthaster planci*, *Diadema setosum* and *Acanthaster planci* (thorn) sp., apoptosis study done. The cells treated with fractions of *Acanthaster planci*, *Diadema setosum*, *Acanthaster planci* (thorn) sp., and vincristine sulfate at the concentrations of IC_50_ 72hr individually. HeLa cells treated with AP-9, APTRN-9, DS-8 and DS-9 fractions were positive to green stain, indicating early apoptotic cells after 3hr incubation. A time-dependent increase in the externalization of phosphatidylserine noticed and detectable by the green stain of annexin V FITC binding. Interestingly, at 24hr treatment, cells were positive to annexin V+ (Early apoptosis), and few cells show PI+ (late apoptosis).

Moreover, the presence of late apoptotic cells in DS-8 and DS-9 was higher than AP-9, and APTRN-9 treated samples, conducted by the presence of red stain of PI conjugated to DNA of treated cells after 24hr (Figure 2 A-D). However, untreated control HeLa cells were viable and negative to annexin V and PI Thus, the results depict that fractions of *Acanthaster planci* and *Diadema setosum* produced apoptosis as a significant cause of the cytotoxicity effects on HeLa cells. 

To further investigate the late apoptosis induced in the HeLa cell line, TUNEL (nick end labelling DNA fragmentation) detection has performed. The cells treated with AP-9, APTRN-9, DS-8 and DS-9 at the concentrations of IC50 at 72hr for 36hr. The induction of DNA fragmentation was detectable via green stain (FITC) of fragmented DNA in fraction treated cells at 36hr. The fluorescence-labelled polymerization of the nucleotide at the fragmented nick ends of DNA via terminal deoxynucleotidyl transferase (TdT). Similarly, control cells were positive to tunnel green nucleus was also detected in the positive control (Vincristine) treated cells. However, nuclei of untreated (control) MCF-7 cells were not positive for green stain when observed under High Content Screening (HCS). Therefore, the results strongly indicate that the fractions induced apoptosis of MCF-7 cells, line via DNA fragmentation.

**Figure 1A:**
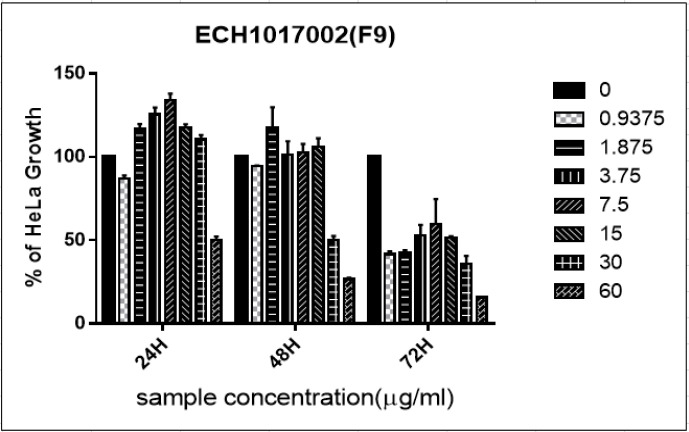
Percentage of Growth Inhibition ± Standard Deviation for Acanthaster Planci AP-9 against HeLa Cells at 24 hr, 48 hr, and 72 hr

**Table 1:** Location and Coordinate where the Samples were Collected

Species	Location of collection
*Acanthaster planci*	Bidong Island, Terengganu (5˚37'12 N 103˚3'48 E)
*Diadema setosum *	Bidong Island, Terengganu (5˚37'12 N 103˚3'48 E)

**Table 2:** IC_50 _Values of Fractions Acanthaster Planci and Diadema Setosum Fractions and Vincristine Sulfate (μg/ml)

	Fractions	IC_50_		
		24 hr	48 hr	72 hr
1	Acanthaster planci AP-9	>60	39.81	3.16
2	Acanthaster planci APTHR-9	>60	28.79	15.84
3	Diadema setosum DS-8	>60	20.33	18.93
4	Diadema setosum DS-9	6.13	3.36	6.89
5	Vincristine Sulphate	-	-	0.005

**Figure 1B:**
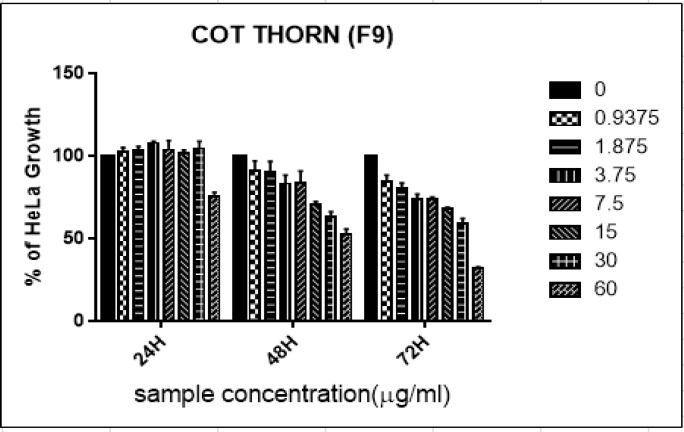
Percentage of Growth Inhibition ± Standard Deviation for Acanthaster Planci (Thorn) APTHR-9 against HeLa Cells at 24 hr, 48 hr, and 72 hr

**Figure 1C:**
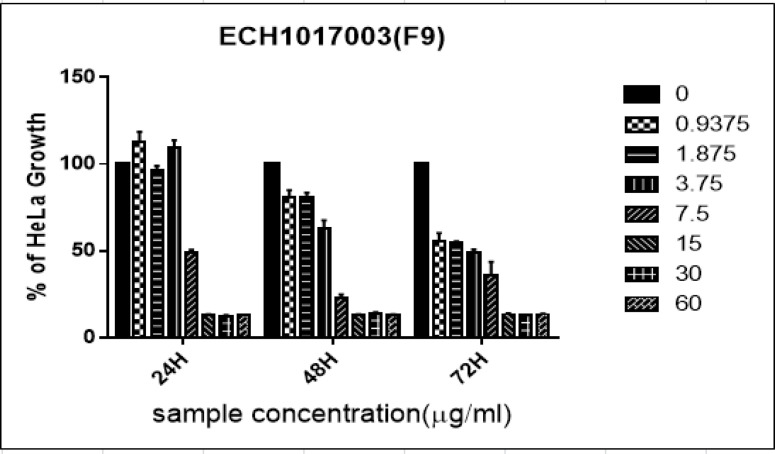
Percentage of Growth Inhibition ± Standard Deviation for Diadema Setosum DS-8 against HeLa Cells at 24 hr, 48 hr, and 72 hr

**Figure 1D:**
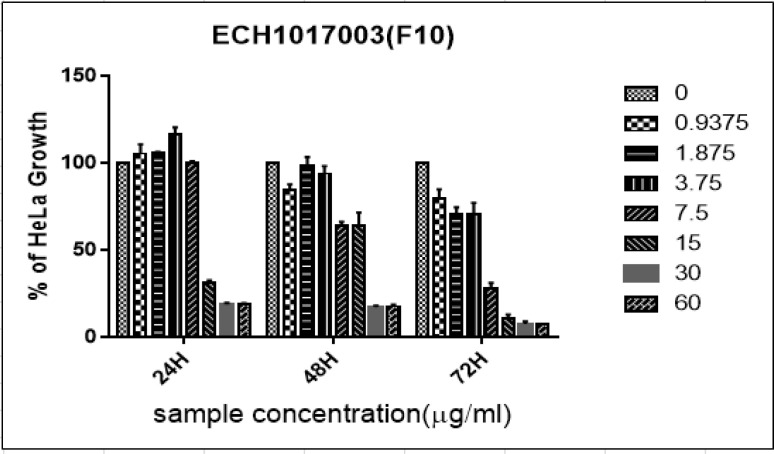
Percentage of Growth Inhibition ± Standard Deviation for Diadema Setosum DS-9 against HeLa Cells at 24hr, 48hr, and 72hr

**Figure 2 (A-E):**
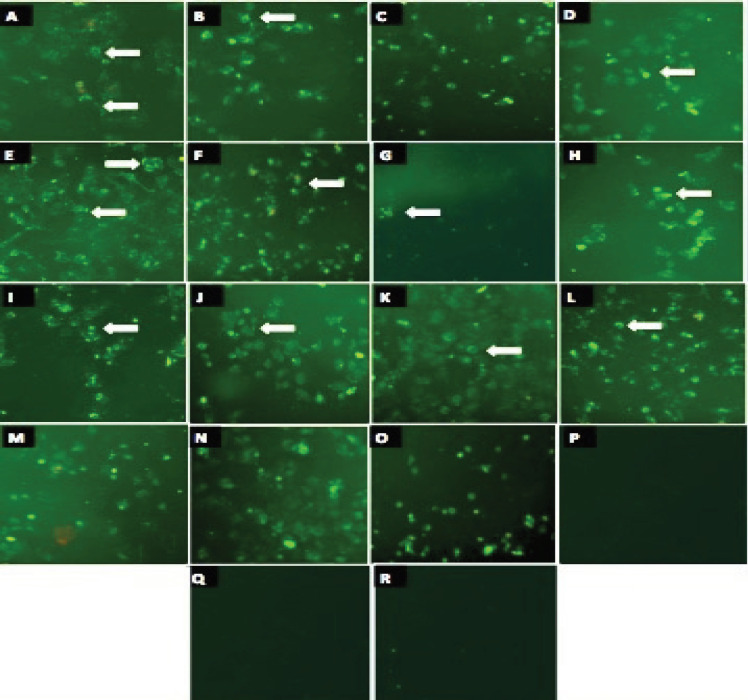
The Green Stain (Annexin-FITC) Early Apoptosis; Red stain, PI (propidium Iodide) late apoptosis indicates induction of apoptosis in HeLa cells; (A,B,C) AP-9 (D,E,F) DS-8 (G,H,I) DS-9 (J,K,L) APTHR (M,N,O) positive control (P,Q,R) Negative control (untreated) for 3hr, 6hr and 24hr respectively

**Figure 3 (A-E):**
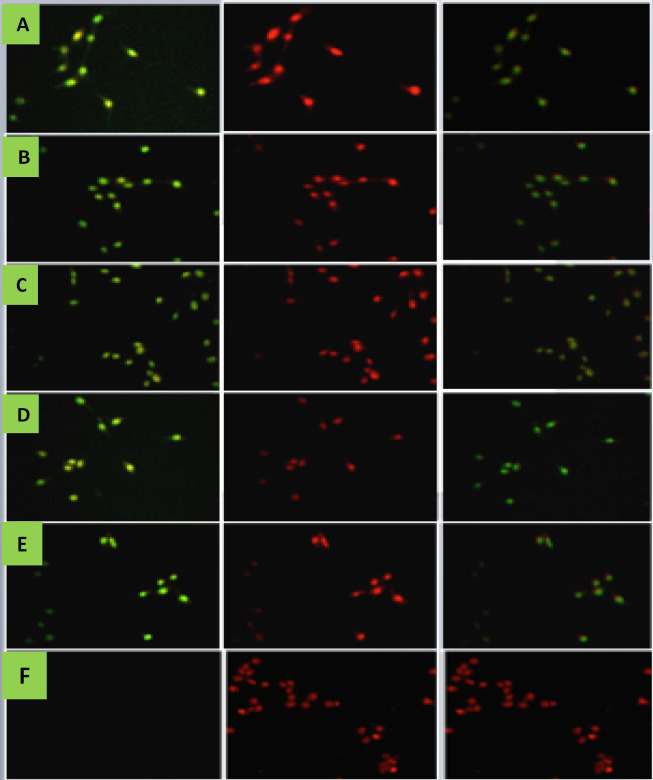
The Presence of Green Stain (FITC), which Indicates DNA Fragmentation in HeLa Cells Treated with Fractions Row A-E. (A) AP-9 (B) APTRN-9 (C) DS-8 (D) DS-9 (E) positive control (Vincristine sulfate) and (F) Negative control (without treatment), after 36 hr

## Discussion

Cancer is uncontrolled cell multiplication or proliferation due to the deregulated process of cell death. The significant criteria of cancer control are tightly regulated cell death and growth. In the last decades, with the continuous increase in the number of cancer cases which includes; acquired resistance, systemic toxicity, which results in the development of secondary cancers. Various side effects such as nausea and vomiting, which creates a significant obstacle in outdoor patient treatment due to the utilization of synthetic drugs, increase the growing interest in developing natural Products base therapeutic agents for cancer treatment (Cragg and Newman, 2013; Sawadogo et al., 2015). Besides, several natural compounds originated or derived from marine life presently undergoing clinical trials with oncological indications (Calado et al., 2018). 

Natural products exhibited novel structures and unique mechanisms of action of marine metabolites, which led to the development of new methods for treating solid tumour types, such as the lung, breast, colon, or prostate (Venter 2004). Moreover, natural products can develop chemotherapy such as vinblastine, vincristine, and Taxol (Gueritte and Fahy 2005; Kingston, 2005). Natural products have unveiled specific and rare chemical structures, which might be insightful for new drugs discovery includes molecular modelling and chemical synthesis (Botana 2015). Among natural products, marine organisms developed distinctive adequate metabolic competencies via quite specific and potent activity compounds (Murray, 2013). 

Therefore, an increasing exploration needed for the screening of marine organisms in the field of anticancer therapeutics. Essentially, the desire for remarkable success has been encouraged by developing cytotoxic active having less toxic abilities of cancer therapeutics (Williams et al., 1989).

Various species of phylum Echinodermata, starfish possessed bioactivities such as antiproliferative, anti-inflammatory and anticancer (Aydın et al., 2011; Conand and Byrne 1995). Certain bioactive secondary metabolites, especially triterpene glycosides, showed remarkable cytotoxic activity. Triterpene glycosides isolated from Pseudocolochirus violaceus showed significant cytotoxic activity against Gastric adenocarcinoma (MKN-45 )and colon carcinoma HCT-116 cell lines (Zhang et al., 2007). Similarly, other metabolites including steroids, steroidal glycosides, saponins, anthraquinones, alkaloids, phospholipids, peptides, and fatty acids were isolated from starfish. These chemical constituents exhibit cytotoxic, hemolytic, antiviral, antifungal, and antimicrobial activities (Dong et al., 2011). 

*Acanthaster planci* extracts and metabolic analogues exerted cytotoxic effects on human breast, lungs, and glioblastoma cell lines. Our study analysed the cytotoxic effects on Hela cells and the mode of cell death, which has not performed before. Previous studies have shown that *Acanthaster planci* starfish extract significantly exhibited potent cytotoxic activity on various cell lines, such as MCF-7 cells, with a low IC_50_ value (15.6 μg/ml). It has also has reported that a saponin analogue, Asterosaponin 1 from the starfish Culcita novaeguineae, induced apoptosis via acquiring powerful cytostatic ability in human glioblastoma U87MG cells (IC_50_ 4.3 μg/ml) and lung cancer A549 cells via dose-dependent manner by inducing apoptosis (Cheng et al., 2006; Zhao et al., 2011). Furthermore, studies signified that the asterosaponins play anticancer agents. Novaeguinoside II, one of the newly found asterosaponin, also has shown to induce apoptosis in U87MG cells by a mitochondrial apoptotic pathway (Zhou et al., 2011). Likewise, the MeOH extract (IC_50_ values ranging from 0.84±0.03 to 3.96±0.14 µg/ml) and asterosaponin (astrosterioside D) from Astropectenmonacanthus exhibited potent in vitro cytotoxic activity against human promyelocytic leukemia cells (HL-60), human prostate cancer cell (PC-3) and colorectal carcinoma cell (SNU-C5) with IC_50_ values ranging from 4.31±0.07 to 5.21±0.15 µM) (Thao et al., 2014). These studies indicated that the asterosaponins play as anticancer agents.

Apoptosis and necrosis are the two foremost mechanisms of cell death (Jan and Chaudhry, 2019). Cancer therapeutic agent induces cell death in cancer cells via mediating apoptosis where the cell lysis occurred regardless of harming normal cells and causing inflammation, which is a characteristic feature of necrosis. Interestingly, in our study, fractions of *Acanthaster planci* and *Diadema setosum* species exerted growth inhibitory or cytotoxicity effects on HeLa cells via apoptosis. Apoptosis tightly defined mechanism of cell death whereby a series of intracellular enzymatic events take place. Each precisely act event of cell death involves features of apoptosis; (i) exposure of phosphatidylserine and (ii) DNA fragmentation. During the early stage of induction of apoptosis, the translocation of phosphatidylserine (PS) from the cytoplasmic side of the membrane to outer surface (Jan and Chaudhry, 2019). Whereas the membrane integrity remains constant, which support the characteristic feature of a cytotoxic agent, which trigger apoptosis and preventing the inflammation or any leakage of intracellular components to extracellular spaces (Fadok 1992).

Our study showed that AP-9, APTRN-9 fraction of *Acanthaster planci* and DS-8 and DS-9 fractions of *Diadema setosum* produced cytotoxicity at the concentrations of 3.16, 15.84, 18.93 and 6.89 μg/ml, respectively subsequently triggered the time-dependent increase in translocation of PS in HeLa cells for 3 hr, 6 hr, and 24hr. The translocation of PS monitored by FITC-labelled-Annexin V has high affinity towards PS, located at the outer membrane of cells (Mahar et al., 2019; Cheng, 2012). Similarly, the confirmation of evidence of apoptosis was done by study the DNA fragmentation. The fragmentation of DNA is the hallmark of apoptosis The TUNEL positive (green) has observed with a fluorescence microscope, which indicated DNA fragmentation. It has reported that steroidal glycosides, Novaeguinoside II extracted from starfish induced DNA fragmentation in human U87MG glioblastoma cells. Novaeguinoside II-induced apoptosis of U87MG cells by a mitochondrial apoptotic pathway increases cyt C and caspase 3 (Zhou et al., 2011). Asterosaponin 1 increased expression and activity of three essential ER-associated apoptotic molecules; CHOP, caspase-4 and JNK results inhibits the proliferation of A549 cells through induction of ER stress-associated apoptosis (Zhao 2011). Therefore, it is tempting to speculate that the compound in the studied fractions used in this study induced apoptosis by similar mechanisms of action. 

This study revealed that AP-9, AP-THR, DS-8, and DS-9 fraction of *Acanthaster planci*, *Diadema setosum* sp., exhibited significant cytotoxicity effects (IC_50_ less than 30 μg/ml at 72hr) on human cervical cancer HeLa cell line. The mode of cell death has analyzed on the basis of phosphatidylserine (PS) exposure (early apoptosis) and in situ DNA fragmentation in cancer cells. Our results showed that fractions exhibited cytotoxicity via induction of apoptosis . The apoptotic mediated cytotoxicity activity might indicate potential metabolites such as alkaloid, flavonoids, and terpenoid compounds. Thus, it could have the beneficial characteristic feature to be candidates for chemotherapeutic drugs to treat cervical cancer. 

## Author Contribution Statement

GS C and MI experimented, GS C performs the apoptosis studies. MI performed the cytotoxicity experiment. MN Z worked on the manuscript analysis. KB performs the fraction analysis, NA A performs the sample collection and treatments, and JS performs the sample identification. YY. S. aided in the implementation of work, and TMT S. supervised the project. GS C wrote the paper with input from all authors.
